# AQP4-AS1 Can Regulate the Expression of Ferroptosis-Related Regulator ALOX15 through Competitive Binding with miR-4476 in Lung Adenocarcinoma

**DOI:** 10.1055/s-0044-1789199

**Published:** 2024-08-16

**Authors:** Lin Du, Geng Xu, Xiuqiang Zhang, Zhiwei Zhang, Yang Yang, Hongsheng Teng, Tao Yang

**Affiliations:** 1 Department of Thoracic Surgery, Tianjin Fifth Center Hospital, Tianjin, China

**Keywords:** lung adenocarcinoma, ALOX15, ferroptosis, AQP4-AS1, miRNA-4476

## Abstract

**Background** The AQP4-AS1/miR-4476-ALOX15 regulatory axis was discovered in previous studies. We aimed to investigate the regulatory mechanism of the ferroptosis-related regulator ALOX15 by AQP4-AS1 and miR-4476 in lung adenocarcinoma (LUAD) and find new targets for clinical treatment.

**Methods** After bioinformatics analysis, we contained one ferroptosis-related gene (FRG), namely ALOX15. MicroRNAs (miRNAs) and long noncoding RNAs were predicted by miRWalk. Furthermore, we constructed overexpressed LUAD cell lines. Real-time quantitative polymerase chain reaction and western blot were used to determine the expression of mRNA and protein, respectively. Cell Counting Kit-8 (CCK-8) and EdU assay were used to detect the cell proliferation. Double luciferase assay was used to detect the binding relationship between AQP4-AS1 and miR-4464.

**Results** ALOX15 was the most significantly downregulated FRG compared with normal tissues. Furthermore, protein-protein interaction network analysis indicated that the AQP4-AS1-miR-4476-ALOX15 regulatory axis might be involved in the occurrence and development of LUAD and there might be direct interaction between AQP4-AS1 and miR-4476, and miR-4476 and ALOX15. Furthermore, AQP4-AS1 and ALOX15 were significantly downregulated in the LUAD tissue and cell lines, whereas miR-4476 showed the opposite results ( *p* < 0.001). AQP4-AS1 overexpression improved the ALOX15 expression in LUAD cell lines. CCK-8 and EdU assay revealed that overexpression of AQP4-AS1 and ALOX15 inhibited the LUAD cell proliferation. Double luciferase assay results indicated that there was a combination between AQP4-AS1 and miRNA-4476. In addition, we found that overexpressed AQP4-AS1 activates the ferroptosis in LUAD cell lines.

**Conclusions** AQP4-AS1 can regulate the expression of ALOX15 through competitive binding with miR-4476, further activate ferroptosis and inhibit the proliferation of LUAD cells.

## Introduction

Lung cancer is the leading cause of cancer-related death. 1 The subtype with the largest proportion of lung cancer is nonsmall cell lung cancer (NCLC), whereas lung adenocarcinoma (LUAD) accounts for the highest proportion in NCLC, reaching 40%. 2 The most common treatment modality for LUAD is surgery, followed by radiotherapy and chemotherapy. 3 However, the above methods have limited therapeutic effect on patients with advanced LUAD. A small number of LUAD patients can benefit from the emerging targeted therapies and immunotherapies in recent years. 4 It is very urgent to find new and efficient LUAD treatments.

The most well-known risk factor for LUAD is smoking, but the increasing number of nonsmoking patients in recent years highlights the role of nonsmoking factors in LUAD. 5 Large-scale genomic studies reveal the presence of LUAD driver genes. 6 Immune checkpoint inhibitor antagonists targeting PD-1 or PD-L1 have been approved for use. However, most LUAD patients still do not benefit from it. 7 Therefore, a deep understanding of the molecular mechanisms of LUAD is crucial for proposing effective therapeutic modalities.

Dysregulation of long noncoding RNAs (lncRNAs) is thought to be associated with a variety of cancers, including LUAD. 8 Aquaporin 4 antisense RNA 1 (AQP4-AS1) transcribes an antisense lncRNA of unknown function. 9 AQP4-AS1 was found to be closely associated with oral squamous cell carcinoma 10 and the overall survival in gastric cancer. 11 Moreover, AQP4-AS1 was reported to be a protective factor for NSCLC. 12 However, in-depth studies on the role of AQP4-AS1 in LUAD are lacking.

MicroRNAs (miRNAs) are endogenous noncoding small RNAs, whose abnormal expression is thought to be associated with a variety of solid tumors. 13 Downregulated miR-198-5p can play a role in the development of LUAD by targeting signaling pathways such as p53. 14 MiRNA-30a-5p can inhibit the proliferation and migration of LUAD cells and is associated with LUAD progression and immune infiltration. 15 The role of miR-4476 in LUAD is rarely reported.

Ferroptosis is a specific mode of cell death driven by iron-dependent phospholipid peroxidation and is involved in a variety of diseases. 16 Studying the pathological mechanism of induction and inhibition of ferroptosis, provides a feasible idea for the treatment of various lipid peroxide-related diseases. 17 Cisplatin-resistant tumor cells are sensitive to ferroptosis. 18 The gene signature associated with ferroptosis is considered as a survival model for predicting the survival of LUAD patients. 19 However, the mechanism of ferroptosis in LUAD remains unclear.

In previous study, we predicted that there was a binding site between AQP4-AS1 and miR-4476, and ALOX15 might be the target gene of miR-4476 in LUAD. In this study, we aimed to validate the relationship between genes in LUAD tumor tissues and cell lines and to explore the correlation with ferroptosis.

## Materials and Methods

### Bioinformatics Analysis

The ferroptosis-related genes (FRGs) collected in this study were downloaded from Gene Set Enrichment Analysis (GSEA, https://www.gsea-msigdb.org ). Gene expression profiles and clinical data from patients with LUAD were downloaded from The Cancer Genome Atlas (TCGA). RNA sequencing data of 525 patients with LUAD and miRNA data of 514 LUAD patients were downloaded from the TCGA database ( https://portal.gdc.cancer.gov/ ). Among them, 502 patients with LUAD have complete corresponding clinical information and were utilized to be further investigated.

The R package limma 14 (version 3.40.6) was used to analyze differentially expressed mRNA, miRNA, and lncRNA among different groups, and mutiMiR15 (version 1.6.0) was used to predict the upstream and downstream mRNA regulated by miRNA and lncRNA. FRGs were downloaded from the Reactome16 website. Factoextra package (version 1.0.7) was used to reduce the dimensions of FRGs expression data and the K-means clustering model was used for clustering analysis. clusterProfiler (version 3.14.3) and DOSE package (version 3.12.0) were used to perform the KEGG (Kyoto Encyclopedia of Genes and Genomes) pathway enrichment analysis and visualization on specific coding mRNA sets. GSEA software 4.1 was used to analyze the enrichment of metabolic gene sets. String website and Cytoscape 7.0 software were used for protein and miRNA interaction analysis.

### Samples Collection

From May 2019 to November 2021, 350 LUAD samples and matched 150 paracancerous samples from the Tianjin Chest Hospital were obtained. Clinical indicators and follow-up data were also collected. Based on the Union for International Cancer Control, the stage of LUAD was assessed. Ethical approval from the hospital and informed consent of the patients were obtained for this study.

### Cell Culture and Transfection

Human LUAD cell lines A549, PC9, and normal lung cell line pulmonary fibroblasts cell were purchased from the ATCC (American Type Culture Collection, United States). The cell lines were cultured in high glucose Dulbecco's modified Eagle medium (Solarbio, China) supplemented with 10% fetal bovine serum (Hyclone, United States) in a 37°C incubator.

Plasmids purchased from Sangon Biotech. Co., Ltd (Shanghai, China) were transfected into cells using lipo3000 (Biosharp, China) according to the manufacturer's instructions. After 24- to 48-hour posttransfection, the transfection efficiency was examined by real-time quantitative polymerase chain reaction (RT-qPCR).

### Cell Counting Kit-8 Assay

The transfected cells were seeded into 96-well plates at a density of 5 × 10 ^3^ cells per well. At 0, 24, 48, and 72 hours after seeding, Cell Counting Kit-8 (CCK-8) reagents were added to 96-well plates and incubated for 1 hour in the dark at a 37°C incubator. A microplate reader was then used to evaluate the absorbance for SOD (450 nm) to calculate cell viability and proliferation rate.

### EdU Assay

The transfected cells were seeded into 24-well plates at a density of 2 × 10 ^5^ cells per well. Then, the EdU solution was added to the cells and the medium was discarded and incubated for 6 to 8 hours. After incubation, cells were washed twice with phosphate-buffered saline (PBS) for 5 minutes each. Finally, a microscope (Leica, Germany) was used to observe the proliferation of cells.

### Real-Time Quantitative Polymerase Chain Reaction Analysis

Total RNA of tissue samples and cells was extracted using RNA Fast kit (Solarbio, China). HiScriptII Reverse Kit (Vazyme, China) was used to reverse transcribe RNA to cDNA. The RT-qPCR was performed using SYBR Green PCR Mix (Biosharp, China) on IQ5 PCR System (Bio-Rad, United States). The PCR program was as follows: 94°C for 1 minute, 94°C for 20 seconds, 58°C for 30 seconds, 72°C for 30 seconds, with 40 cycles. Information on primers used in this study was shown in Supplementary Table S1 (available in online version only). The relative expression of genes was calculated with 2 ^−ΔΔCt^ .

### Western Blotting

Cells were resuspended in 0.5 mL RIPA (radioimmunoprecipitation assay) lysis buffer on ice for 30 minutes. Then, the proteins subjected to centrifugation at 10,000 g for 20 minutes were collected to concentration determination using the BAC kit (Thermo Scientific, United States). Protein samples were then electrophoresed using SDS-PAGE (sodium dodecyl-sulfate polyacrylamide gel electrophoresis) and transferred to polyvinylidene difluoride membranes. Subsequently, samples were blocked with 5% nonfat milk in tris-buffered saline Tween-20 solution at room temperature for 2 hours and then probed with ALOX15 and Tubulin primary antibody (Boster, China, dilution: 1:2000) at 4°C overnight. Secondary antibodies used for detection were horseradish peroxidase-conjugated anti-rabbit IgG. Finally, an ECL (Excellent Chemiluminescent Substrate) chemiluminescence detection kit (Boster, China) was used to detect the expression of proteins.

### Double Luciferase Reporter Assay

Twenty-four hours after cells and plasmids were cotransfected, the medium was discarded. Then, 5× passive lysis buffer (PLB) was then diluted with deionized water to 1× PLB and added to the cells. Fifteen minutes later, the lysate was centrifuged for 10 minutes with 13,200 g, and the supernatant was collected. Ninety-six-well plates with 10 μL of supernatant per well were added with 100 μL of premixed luciferase reagent to detect the luciferase reaction intensity. After the detection was completed, 150 μL of stop reagent premixed was added to each well for 5 seconds. Then, the data were measured to determine the intensity of the internal reference Renilla luciferase reaction. The ratio of the two sets of data was calculated.

### Lipid Peroxidation and Reactive Oxygen Species Assay

The content of malondialdehyde (MDA) was used to assess the lipid peroxidation followed the manufacturer's instructions. The kit for the determination of MDA was purchased from Nanjing Jiancheng Institute of Bioengineering, China (catalog number: A003-1-2).

The fluorescent probe 2,7-dichlorodi-hydrofluorescein diacetate (DCFH-DA) was used to determine the reactive oxygen species (ROS) levels. Specifically, the transfected cells were centrifuged for 10 minutes with 2500 r/min and washed with PBS. Then, the cells were stained with 10 μg mL-1 DCFH-DA at 37°C for 30 minutes in the dark. Finally, the stained cells were collected and washed with PBS. The fluorescence density was measured using a fluorescence microplate detector (Bio-Tek, United States) with an excitation wavelength of 488 nm and an emission wavelength of 520 nm.

### Detection of the Iron Content in the Transfected Cell

Transfected cells were centrifuged at 2500 r/min for 10 minutes. A total of 100 mL of distilled water and 2 mg/L of iron standard solution were added to the supernatant. Subsequently, 300 mL of iron developer was added. Five minutes later, the mixed solution was centrifuged at 3500 r/min for 10 minutes. Finally, 200 mL of supernatant was collected and detected for absorbance at 520 nm. Iron content 3 (mg/gprot) = (determined OD value − blank OD value)/(standard OD value − blank OD value) × standard concentration (2 mg/L)/protein concentration of the sample to be tested (gprot/L).

### Detection of the GSH/GSSG and GPX

Transfected cells were centrifuged at 12,000g for 10 minutes and the supernatant was collected. The standards were diluted in lysis buffer to 0.01, 0.03, 0.1, 0.3, 1, 3, and 10 µM concentrations, respectively. A total of 100 µL of the working solution was added to a 96-well plate and left at room temperature for 5 minutes to deplete background. Finally, RLU (Relative Light Unit) was measured with a chemiluminescence analyzer 2 seconds after the addition of 20 µL of sample or standard.

### Statistical Analysis

GraphPad Prism 5.0 was used for data statistical analysis. The data were expressed as mean ± standard deviation. The difference between two groups was compared using *t* -test, whereas multiple groups were compared using one-way analysis of variance test. *p* < 0.05 was considered as statistically significant.

## Results

### Differences in Ferroptosis-Related Genes between the Lung Adenocarcinoma and Healthy Population Groups

**Fig. 1:**
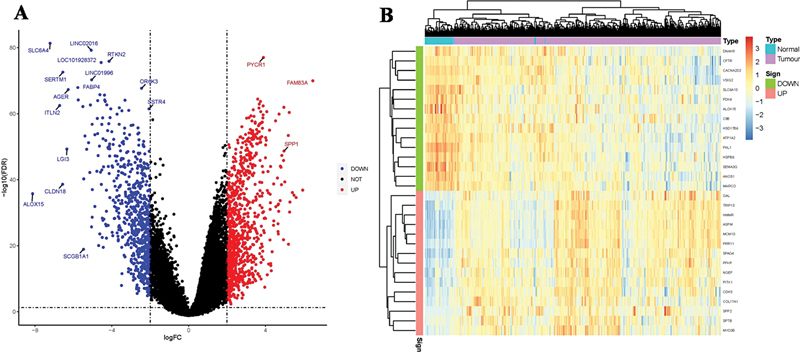
ALOX15 was the most significantly downregulated FRG in LUAD tissues. ( **A** ) Volcano map. ( **B** ) Heatmap. FRG, ferroptosis-related gene; LUAD, lung adenocarcinoma.

**Fig. 2:**
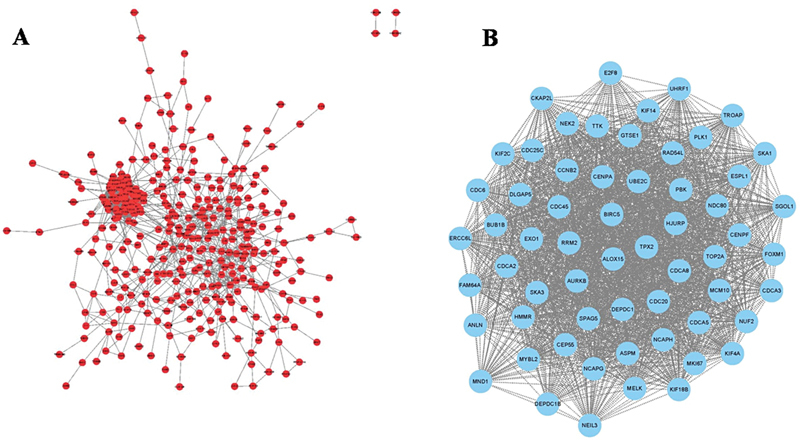
PPI analysis of AQP4-AS1-miR-4464-ALOX15 regulatory axis. (A) AQP4-AS1-miR-4464 regulatory network. (B) miR-4464-ALOX15 regulatory network.

From the volcano map and heatmap, we identified a total of 34 differentially expressed FRGs between LUAD and normal tissues, and ALOX15 was the most significantly downregulated FRG in LUAD tissues compared with normal tissues ( Fig. 1A, B ). Furthermore, protein-protein interaction network analysis indicated that the AQP4-AS1- miR-4476-ALOX15 regulatory axis might be involved in the occurrence and development of LUAD and there might be direct interaction between AQP4-AS1 and miR-4476, and miR-4476 and ALOX15 ( Fig. 2A, B ). The specific molecular mechanism could need to be verified by further experiments.

### The Expression of AQP4-AS1, miR-4476, and ALOX15 in the Lung Adenocarcinoma Tissues and Cell Lines

**Fig. 3:**
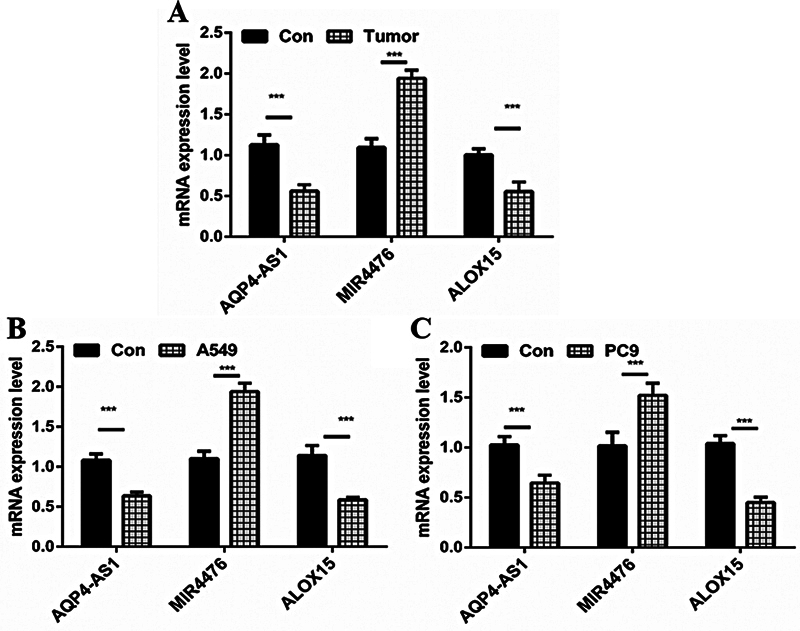
The relative expression of AQP4-AS1, miR4476, and ALOX15. ( **A** ) The relative expression of AQP4-AS1, miR4476, and ALOX15 in LUAD and paracancer tissues. ( **B** ) The relative expression of AQP4-AS1, miR4476, and ALOX15 in LUAD cell line A549 and normal cell line PFC. ( **C** ) The relative expression of AQP4-AS1, miR4476, and ALOX15 in LUAD cell line PC9 and normal cell line PFC. *** represents *p* < 0.001. LUAD, lung adenocarcinoma; PFC, pulmonary fibroblasts cell.

In a previous study, we found a correlation between the expression level of AQP4-AS1 and miR-4476/ALOX15 by bioinformatics analysis. Here, we validated the expression of the above three genes using LUAD tumor tissue and two cell lines. As shown in Fig. 3A , in tumor tissues, the expression levels of AQP4-AS1 and ALOX15 were significantly lower than those in normal tissues ( *p* < 0.001), whereas miR-4476 showed the opposite results ( *p* < 0.001). This is consistent with our predictions. Similarity, in the LUAD cell line A549 ( Fig. 3B ) and PC9 ( Fig. 3C ), the detection results of the expression levels of the three genes (AQP4-AS1, miR-4476, ALOX15) were consistent with the detection results in tumor tissue ( *p* < 0.001). The cell lines A549 and PC9 were used in subsequent experiments.

### Overexpression of AQP4-AS1 Improves the Expression of Ferroptosis-Related Regulator ALOX15 in Lung Adenocarcinoma Cell Lines

**Fig. 4:**
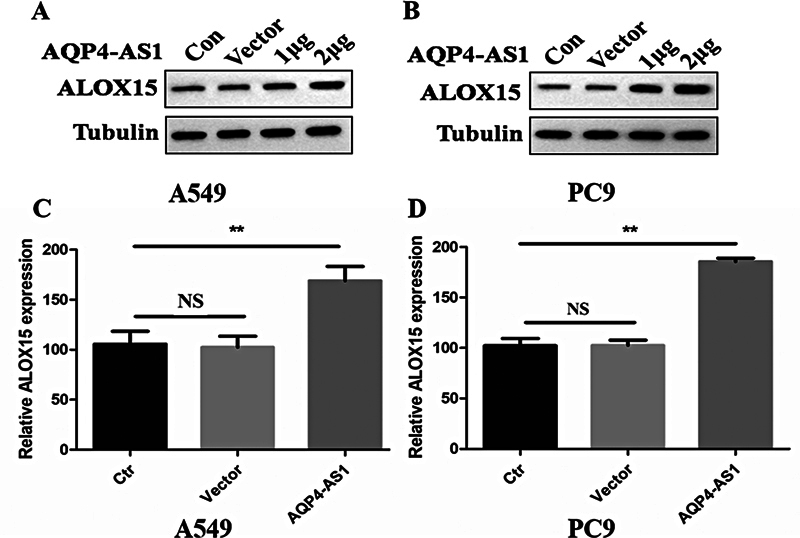
Relative expression of ALOX15 in the AQP4-AS1 overexpressed LUAD cells. ( **A** ) Relative expression of protein ALOX15 in the AQP4-AS1 overexpressed LUAD cell line A549. ( **B** ) Relative expression of protein ALOX15 in the AQP4-AS1 overexpressed LUAD cell line PC9. ( **C** ) Relative expression of mRNA ALOX15 in the AQP4-AS1 overexpressed LUAD cell line A549. ( **D** ) Relative expression of mRNA ALOX15 in the AQP4-AS1 overexpressed LUAD cell line PC9. Con and Ctr represent the control LUAD cell lines. Vector represents the LUAD cell lines, which is transfected with an empty vector. ** represents *p* < 0.01. LUAD, lung adenocarcinoma.

In order to study the function of AQP4-AS1 on ferroptosis-related regulator ALOX15 in LUAD, we transfected the vector-AQP4-AS1-OE into the PC9 and A549 cell lines. In the PC9 cell line ( Fig. 4A, C ), the expression of ALOX15 was significantly increased in the AQP4-AS1-OE group compared with the control and empty vector groups ( *p* < 0.01). In addition, we detected the expression of ALOX15 in the A549 cell lines ( Fig. 4B, D ). The result indicated that in the AQP4-AS1-OE group, the expression of ALOX15 was significantly upregulated than that in the control and empty vector groups ( *p* < 0.01).

### Overexpression of AQP4-AS1 and ALOX15 Inhibit the Proliferation of Lung Adenocarcinoma Cells

**Fig. 5:**
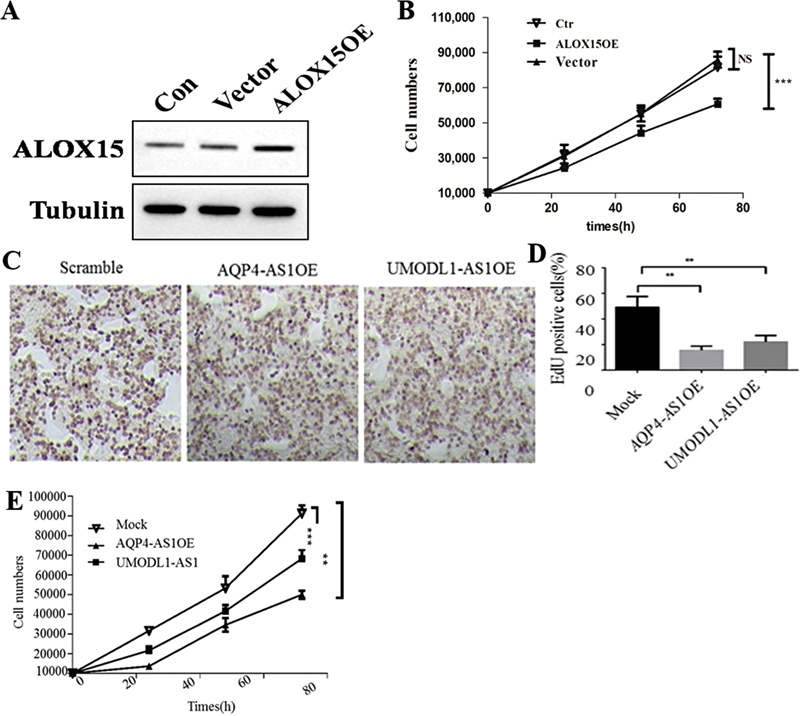
Overexpression of AQP4-AS1 and ALOX15 inhibits LUAD cell proliferation. ( **A** ) The relative expression of protein ALOX15 in A549 and ALOX15-overexpression cells. ( **B** ) Detection of A549 cells' proliferation by CCK8 assay. ( **C** ) Detection of A549 cells proliferation by EdU assay. ( **D** ) Statistics of EdU positive A549 cell rate. ( **E** ) Detection the proliferation level of A549 cells in a time-dependent manner by EdU assay. Con and Ctr represents the control LUAD cell line A549. Vector represents the LUAD cell line A549, which transfected with empty vector. Scramble and mock represent the control LUAD cell line A549. UMODL1-AS1 represents the positive control. ** and *** represent *p* < 0.01 and 0.001, respectively. LUAD, lung adenocarcinoma.

To assess the function of ALOX15 in LUAD, we transfected the vector-ALOX15-OE in the A549 cell line. The results of WB assay showed that the expression level of ALOX15 in the ALOX15-OE group was higher than that in the control and empty vector groups ( Fig. 5A ). Further CCK-8 assay indicated that the cell viability ratio of ALOX15-OE group was significantly lower than that of the control and empty vector groups ( Fig. 5B ). In addition, we used EdU assay to examine the effect of AQP4-AS1 on the proliferation of LUAD cells. The results showed that the proliferation of cells in the AQP4-AS1-OE group was significantly inhibited, compared with the scramble and positive control UMODL1-AS1-OE groups ( Fig. 5C–E ).

### Combination between AQP4-AS1 and miRNA-4476

**Fig. 6:**
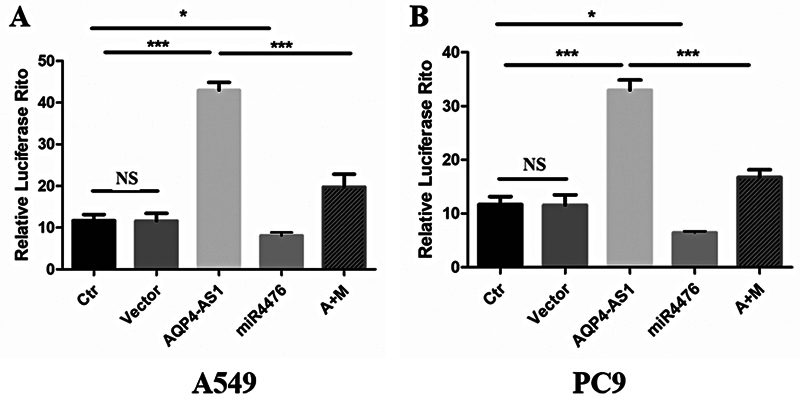
Demonstrating the interaction relationship of miR4476 and AQP4-AS1. ( **A** ) Detection of miR4476 and AQP4-AS1 interaction in LUAD cell line A549 by dual luciferase assay. ( **B** ) Detection of miR4476 and AQP4-AS1 interaction in LUAD cell line PC9 by dual luciferase assay. Ctr represents the control LUAD cell line A549 or PC9. Vector represents the LUAD cell line A549 or PC9, which is transfected with an empty vector. *, **, and *** represent *p* < 0.05, 0.01, and 0.001, respectively. LUAD, lung adenocarcinoma.

Our previous study found a binding site between AQP4-AS1 and miRNA-4476. To further verify the result, we performed a dual luciferase assay in A549 and PC9 cell lines. As shown in Fig. 6A , the relative luciferase ratio of the AQP4-AS1 group was significantly higher than that of the control group ( *p* < 0.001), while that of the miR-4476 group was significantly lower than that of the control group ( *p* < 0.05). Furthermore, we also found that the relative ratio of luciferase in the AQP4-AS1 + miR-4476 group was significantly lower than that in the AQP4-AS1 group ( *p* < 0.001). Similar results were obtained in PC9 cell line as in A549 cell line ( Fig. 6B ). In conclusion, we confirmed a binding relationship between AQP4-AS1 and miRNA-4476 by dual luciferase assay.

### Ferroptosis is Involved in Lung Adenocarcinoma In Vivo and In Vitro

**Fig. 7:**
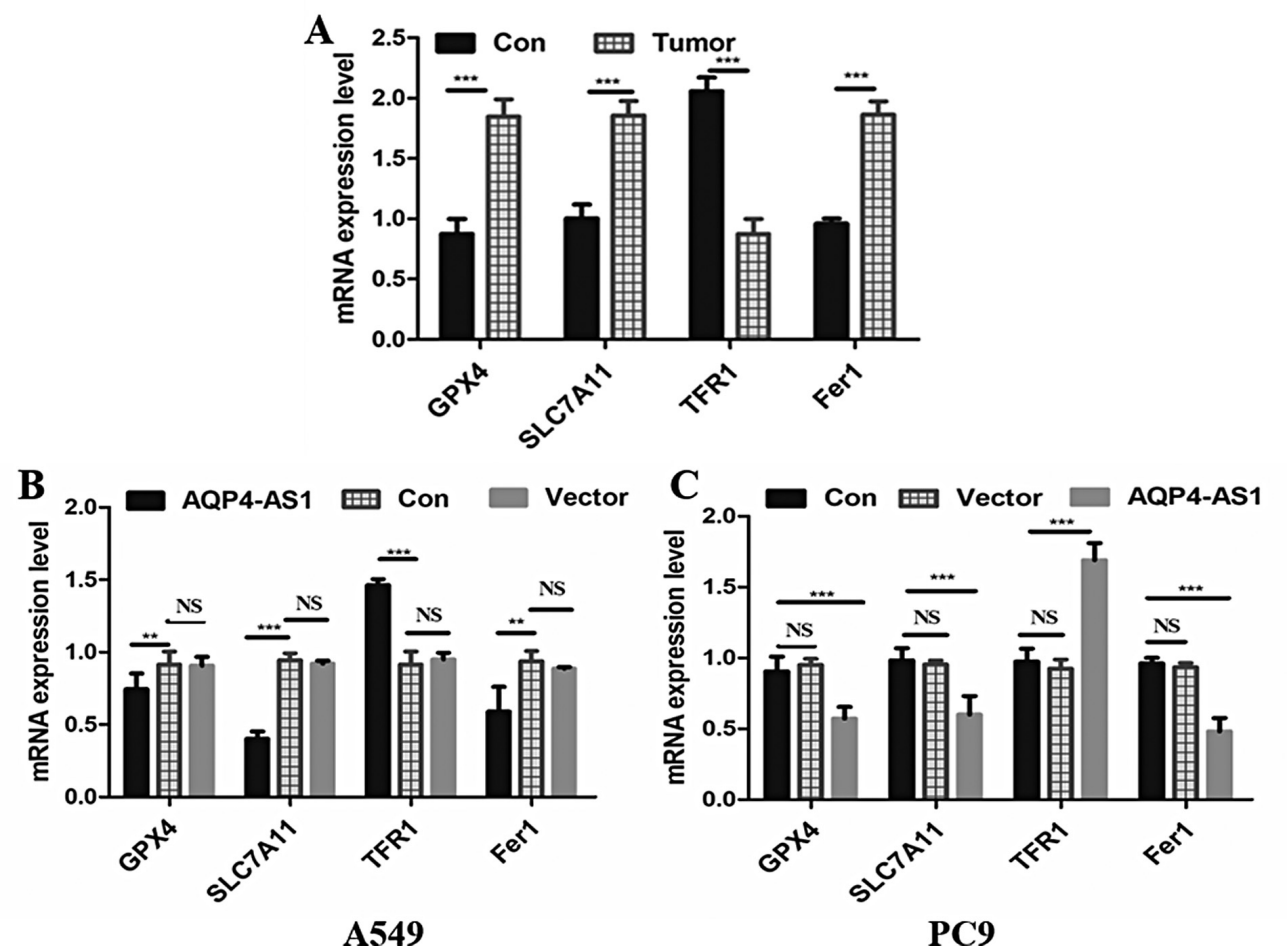
Relative expression of ferroptosis-related genes in the LUAD tissues and cell lines. ( **A** ) Relative expression of ferroptosis-related genes in the LUAD and paracancer tissues. ( **B** ) Relative expression of ferroptosis-related genes in the LUAD cell line A549. ( **C** ) Relative expression of ferroptosis-related genes in the LUAD cell line PC9. Con represents paracancer tissues, the LUAD cell line A549 or PC9. Vector represents the LUAD cell line A549 or PC9, which is transfected with an empty vector. ** and *** represent *p* < 0.01 and 0.001, respectively. LUAD, lung adenocarcinoma.

To investigate the role of ferroptosis in LUAD, we examined the expression of FRGs in LUAD tissues and paracancerous tissues ( Fig. 7A ). Compared with normal tissues, the relative expression of GPX4, SLC7A11, and Fer1 was significantly upregulated in tumor tissues ( *p* < 0.001). The relative expression of TFR1 in tumor tissues was significantly downregulated than that in adjacent tissues ( *p* < 0.001).

In addition, we also examined the expression of FRGs in AQP4-AS1-overexpressed LUAD cell lines ( Fig. 7B, C ). Both in the A549 and PC9 cells, the relative expression of GPX4, SLC7A11, and Fer1 was significantly downregulated in the AQP4-AS1-overexpressed group compared with the control and empty vector groups ( *p* < 0.001). The relative expression of TFR1 was significantly upregulated in the AQP4-AS1-overexpressed group compared with the control and empty vector groups ( *p* < 0.001). This result indicated that overexpression of the AQP4-AS1 suppressed the expression of GPX4, SLC7A11, and Fer1 in LUAD cell lines but promoted the expression of TFR1 in LUAD cell lines. In conclusion, ferroptosis plays a role in LUAD and is regulated by AQP4-AS1.

### Overexpressed AQP4-AS1 Activates the Ferroptosis in Lung Adenocarcinoma Cell Lines

**Fig. 8:**
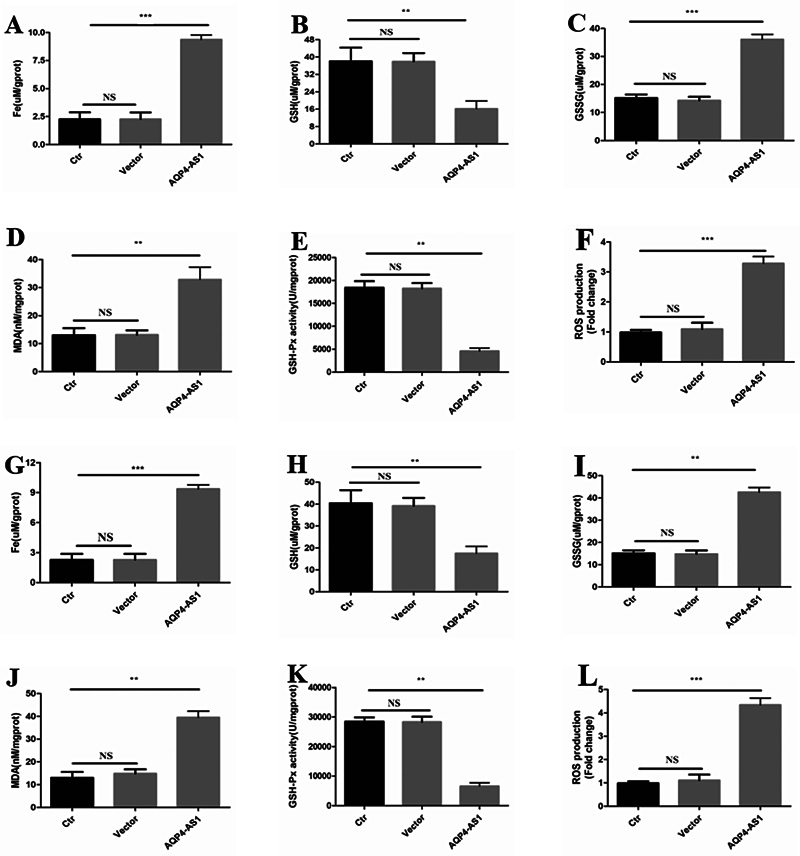
Detection of ferroptosis-related indicators. ( **A, B, C, D, E** , and **F** ) represent the level of Fe, GSH, GSSG, MDA, GSH-Px, and ROS in the LUAD cell line A549. ( **G, H, I, J, K** , and **L** ) represent the level of Fe, GSH, GSSG, MDA, GSH-Px, and ROS in the LUAD cell line PC9. Ctr represents the control LUAD cell line A549 or PC9. Vector represents the LUAD cell line A549 or PC9, which is transfected with an empty vector. ** and *** represent *p* < 0.01 and 0.001, respectively. LUAD, lung adenocarcinoma; ROS, reactive oxygen species.

To determine whether overexpression of AQP4-AS1 is the activation of ferroptosis in LUAD cell lines, the ferroptosis-related indicators in AQP4-AS1 overexpression cell lines were detected ( Fig. 8 ). The results indicated that the Fe, GSSG, MDA, and ROS levels in the AQP4-AS1 overexpressed A549 cells were significantly higher than that in the control and empty vector groups ( Fig. 8A–D , *p* < 0.01). The GSH and GSH-Px levels in the AQP4-AS1 overexpressed A549 cells were significantly lower than that in the control and empty vector groups ( Fig. 6E, F , *p* < 0.01).

We also detected ferroptosis-related markers in AQP4-AS1 overexpressed PC9 cells. The detection results in PC9 cells were consistent with the detection results in A549 cells ( Fig. 8G–L , *p* < 0.01). The above results suggested that overexpression of AQP4-AS1 can activate ferroptosis in LUAD cells.

## Discussion

LUAD is one of the deadliest diseases in humans. According to statistics, 70% of LUAD patients will develop tumor metastasis. 20 In recent years, the continuous development of third-generation drugs and molecularly targeted drugs has benefited a small number of patients. Most LUAD patients still face poor treatment outcomes. 21 The further development of highly effective targeted therapies has become a hot topic in cancer treatment in recent years. Ferroptosis may be relevant to a number of physiological and pathophysiological conditions of many diseases, including LUAD. Moreover, triggering ferroptosis enhanced radiosensitivity in human patient-derived models of LUAD. Previous studies identified FRG signature in LUAD using bioinformatics analyses. However, poor research indicated the regulation of noncoding RNA associated with ferroptosis. In this study, we constructed a competing endogenous RNA network via screening miRNAs and lncRNAs associated with FRGs that could provide the underlying mechanisms to improve the long-term survival of LUAD patients. At the same time, by detecting the expression of AQP4-AS1, miR-4476, and ALOX15 in LUAD tissues and cancer cell lines, it was proved that AQP4-AS1 and miR-4476 were significantly highly expressed in LUAD tissues and cancer cell lines, whereas ALOX15 was expressed significantly high in normal cells. This is consistent with the results of our previous bioinformatics analysis.

In further studies, we verified whether the expression of ALOX15 is regulated by the expression of AQP4-AS1. Through transfection experiments, we found that overexpression of AQP4-AS1 could significantly increase the expression of ALOX15 in LUAD cell lines. Then, we analyzed the biological effects of AQP4-AS1 and ALOX15 on LUAD cells. We found that overexpression of ALOX15 inhibited LUAD cell proliferation. Further study demonstrated that overexpression of AQP4-AS1 also inhibited LUAD cell proliferation. A previous study reported that AQP4-AS1 was a protective factor for NSCLC. 12 ALOX15 is a ferroptosis driver gene involved in the pathogenesis of human diseases including cardiovascular and metabolic diseases. 22 Downregulation of ALOX15 enhances the link between colitis and colorectal carcinogenesis. 23 Ren et al constructed a signature of ten genes including ALOX15 as a potential novel immunotherapy biomarker for studying LUAD. 24 These results suggested that AQP4-AS1 and ALOX15 play important roles in LUAD.

In a previous study, we predicted the existence of a binding site between AQP4-AS1 and miRNA-4476. Dual luciferase assay was used to verify the above hypothesis. Based on the above results, we propose that AQP4-AS1 plays a role in LUAD by regulating the expression of ALOX15 through competitive binding to miRNA-4476.

Since ALOX15 is a ferroptosis driver gene, we aimed to explore whether the regulation of ALOX15 in LUAD by AQP4-AS1 and miRNA-4476 involves ferroptosis. We detected the expression of FRGs in LUAD tissues and normal tissues. The results showed that the expression of GPX4 in tumor tissues was significantly higher than that in normal tissues. This is consistent with a previous related report. 25 Study has reported that GPX4 regulates ferroptosis caused by 12 different compounds, and its low expression induces ferroptosis and inhibits the proliferation of renal cell carcinoma cell lines. 26 SLC7A11 also showed high expression in tumor tissues in this study. It is found that overexpression of SLC7A11 can promote tumor growth by inhibiting ferroptosis. 25 SLC7A11 was shown to be overexpressed in LUAD patients and promote tumor progression. 27 Fer-1 was shown to be highly expressed in LUAD tissues in this study. Previous study has demonstrated that Fer-1 downregulates the level of ferroptosis in lung tissue and has a therapeutic effect on lipopolysaccharide-induced acute lung injury. 28 TFR1 is the major protein required for iron uptake. Data in the TIMER database show that TFR1 is downregulated in LUAD tissues. 29 This is consistent with our findings. In addition, we verified the effect of AQP4-AS1 overexpression on the expression of FRGs. The results showed that overexpression of AQP4-AS1 decreased the expression of GPX4, SLC7A11, and Fer-1 in LUAD cell lines, while increased the expression of TFR1 in LUAD cell lines.

Ferroptosis is suppressed in tumor development. 24 The ferroptosis process inhibits tumor development in LUAD. 30 We detected ferroptosis-related indicators in AQP4-AS1-overexpression LUAD cell lines. We found higher levels of Fe, GSSG, MDA, and ROS in AQP4-AS1-overexpression LUAD cell lines. Previous study has found that the activation of ferroptosis is accompanied by the production of ROS and the accumulation of MDA and Fe. 31 We also found that GSH and GSH-Px levels were significantly reduced in AQP4-AS1 overexpression LUAD cells. The ferroptosis process is often accompanied by an imbalance of GSH and GSSG. 31 Our study is the first to demonstrate that AQP4-AS1 can activate ferroptosis in LUAD cells.

In summary, our study demonstrated that AQP4-AS1 can regulate the expression of ferroptosis-related regulator ALOX15 through competitive binding with miR-4476, further activating ferroptosis process and inhibiting the proliferation of LUAD tumor cells. AQP4-AS1 as a potential LUAD therapeutic target needs to be further investigated in more in vivo and in vitro experiments.
