# Supplementary material for: AQP4-AS1 Can Regulate the Expression of Ferroptosis-Related Regulator ALOX15 through Competitive Binding with miR-4476 in Lung Adenocarcinoma
Source: Glob Med Genet. 2024 Aug 16;11(4):241–50. doi: 10.1055/s-0044-1789199 (PMC11329318; doi:10.1055/s-0044-1789199)
Supplement: Supplementary file 1 — Supplementary Material [file 10-1055-s-0044-1789199-s2400056.pdf]

Supplementary Table S1 Primers information

Primer	Forward sequence (5′–3′)	Reverse sequence (5′–3′)
AQP4-AS1	GTCAATACGGATCATATCCT	ACTGCAACTGACCATTCTAC
miR-4464	AGCAGTTTATTGACATAAATCAA	CGTGAACTCGGTCTATGATTCA
ALOX15	AACTTTCGCTTAGTGGAACGT	ACCCTCATACCTTTGGAACAG
SLC7A11	CTCCTCTGCATTGCCATTGT	TGTGGCTCGAGGTATTGTCA
GPX4	CGACAAGCCTCCCAGGTTCA	GTGCCACCCAGCCAGCTATC
TFR1	CCTGCAAATCGCTCATAGAC	GAGTTCCTAAGCTACACAA
Fer-1	ACCACAACAGCAAGACAC	ATGACGAGACGATAACCCTT
U6	CTCGCTTCGGCAGCACA	ACGCTTCACGAATTTGC
GAPDH	AGAAGGCTGGGGCTCATTTG	AGGGGCCATCCACAGTCTTC

 © 2024. The Author(s).
